# Quadrigeminal Cistern Lipoma: A Case Report

**DOI:** 10.7759/cureus.113990

**Published:** 2026-08-05

**Authors:** Chaimaa Amry, Abd El Hamid Jehri, Khadija Ibahioin, Said Hilmani, Abdelhakim Lakhdar

**Affiliations:** 1 Neurosurgery, Centre Hospitalier Universitaire Ibn Rochd, Casablanca, MAR

**Keywords:** discovered incidentally, intracranial lipomas, radiological findings, rare location, treatment choices

## Abstract

Intracranial lipomas are congenital malformations resulting from abnormal differentiation of the primitive meninx. They are most often discovered incidentally during neuroimaging performed for unrelated conditions. We report the case of an 11-year-old child who underwent brain magnetic resonance imaging (MRI) following head trauma without associated neurological symptoms. Imaging revealed a 1.3-cm right paramedian lipoma located within the quadrigeminal cistern, extending into the cerebral aqueduct without evidence of hydrocephalus. Given the absence of clinical manifestations, conservative management with radiological follow-up was adopted. Intracranial lipomas are typically slow-growing and asymptomatic lesions, most commonly found in the pericallosal cistern, followed by the quadrigeminal cistern. Although rare symptomatic cases may present with headaches, seizures, hydrocephalus, or visual disturbances requiring medical or surgical treatment, surgical intervention is generally avoided because of the intimate relationship of these lesions with critical neurovascular structures and their benign natural history. This case highlights the importance of recognizing intracranial lipomas as incidental findings and supports conservative management in asymptomatic patients, for whom the risks of surgery usually outweigh the potential benefits.

## Introduction

Intracranial lipomas (IL) are rare lesions, accounting for only 0.1% to 0.5% of all intracranial tumors. First described by Meckel in 1813, intracranial lipomas are considered congenital malformations that arise from the abnormal development and persistence of the primitive meninges, typically located along the midline [1,2]. Intracranial lipomas are most frequently detected incidentally, as illustrated in our clinical case. They are contingent upon their location, and they are predominantly asymptomatic. They are typically identified during computed tomography (CT) scans or magnetic resonance imaging (MRI) conducted for alternative clinical indications, such as head trauma or headaches [3]. Identifying these lesions early is crucial for effective management and improved patient outcomes. They rarely necessitate surgical intervention.

## Case presentation

**Figure 1:**
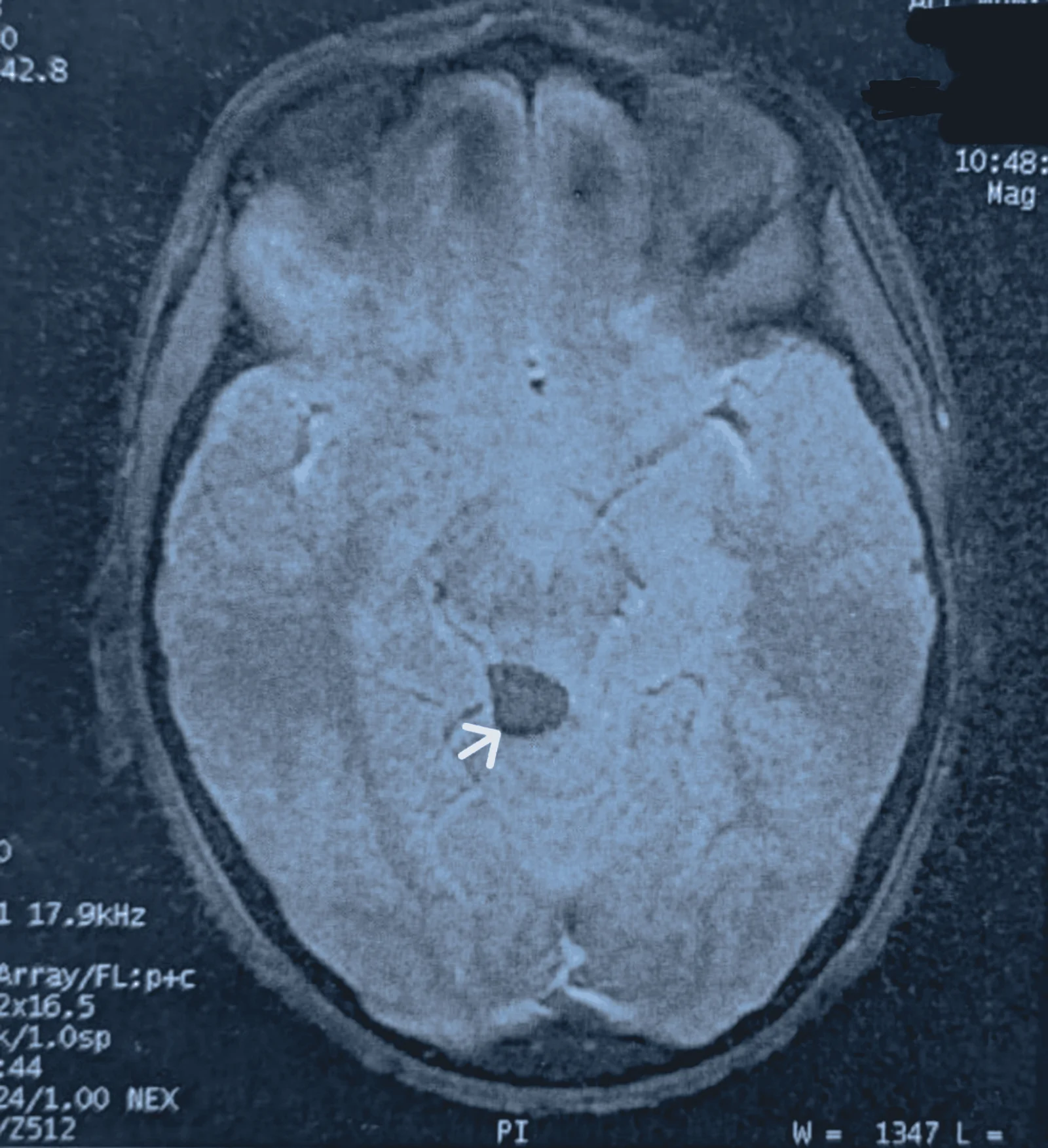
Axial T1 FAT SAT (fat-saturated) images magnetic resonance imaging brain image showing quadrigeminal cistern lipoma on the right side (white arrow)

**Figure 2:**
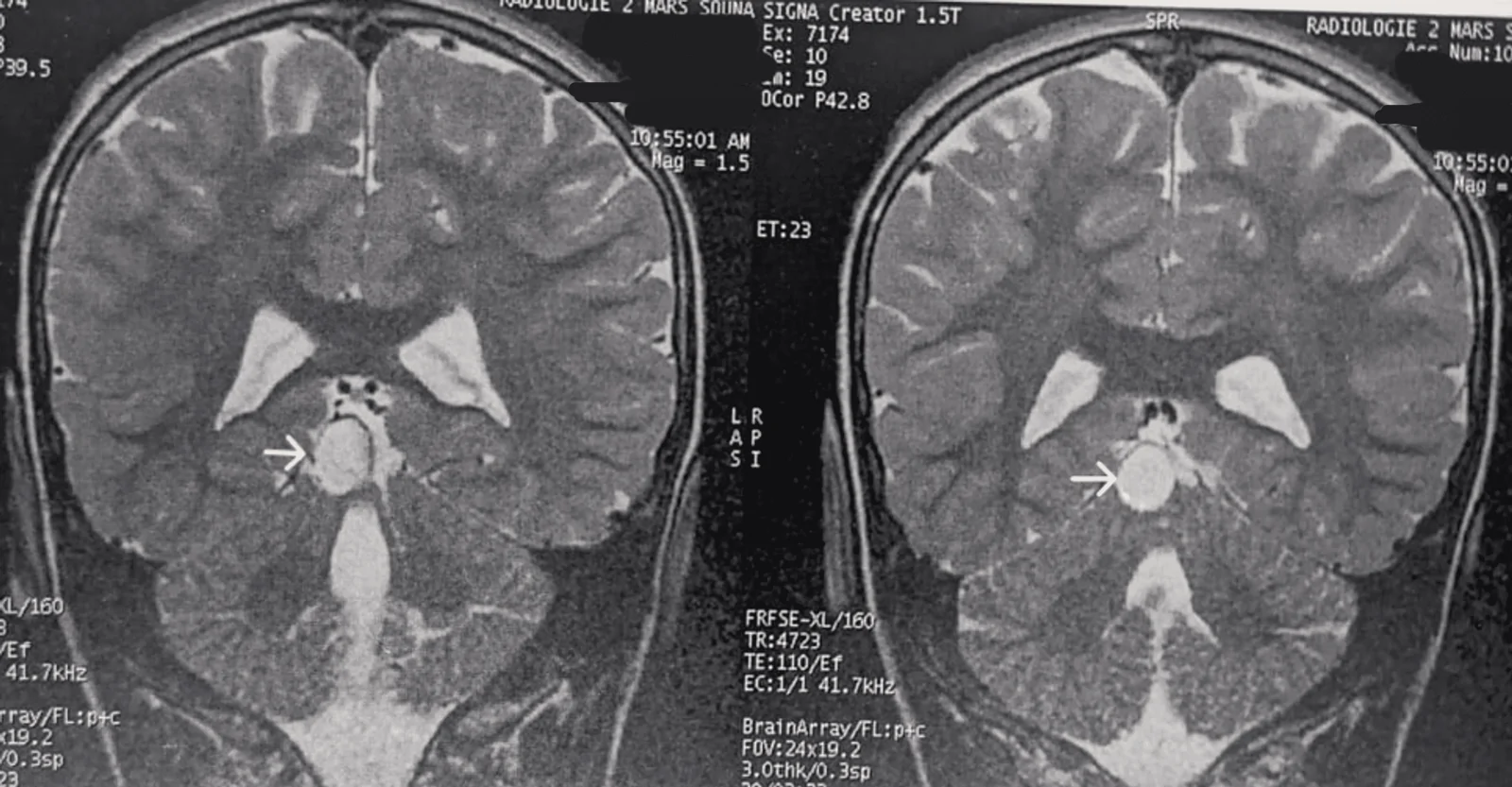
Coronal T2 brain image showing T2 hyperintense lesion in the quadrigeminal cistern on the right side (white arrows)

**Figure 3:**
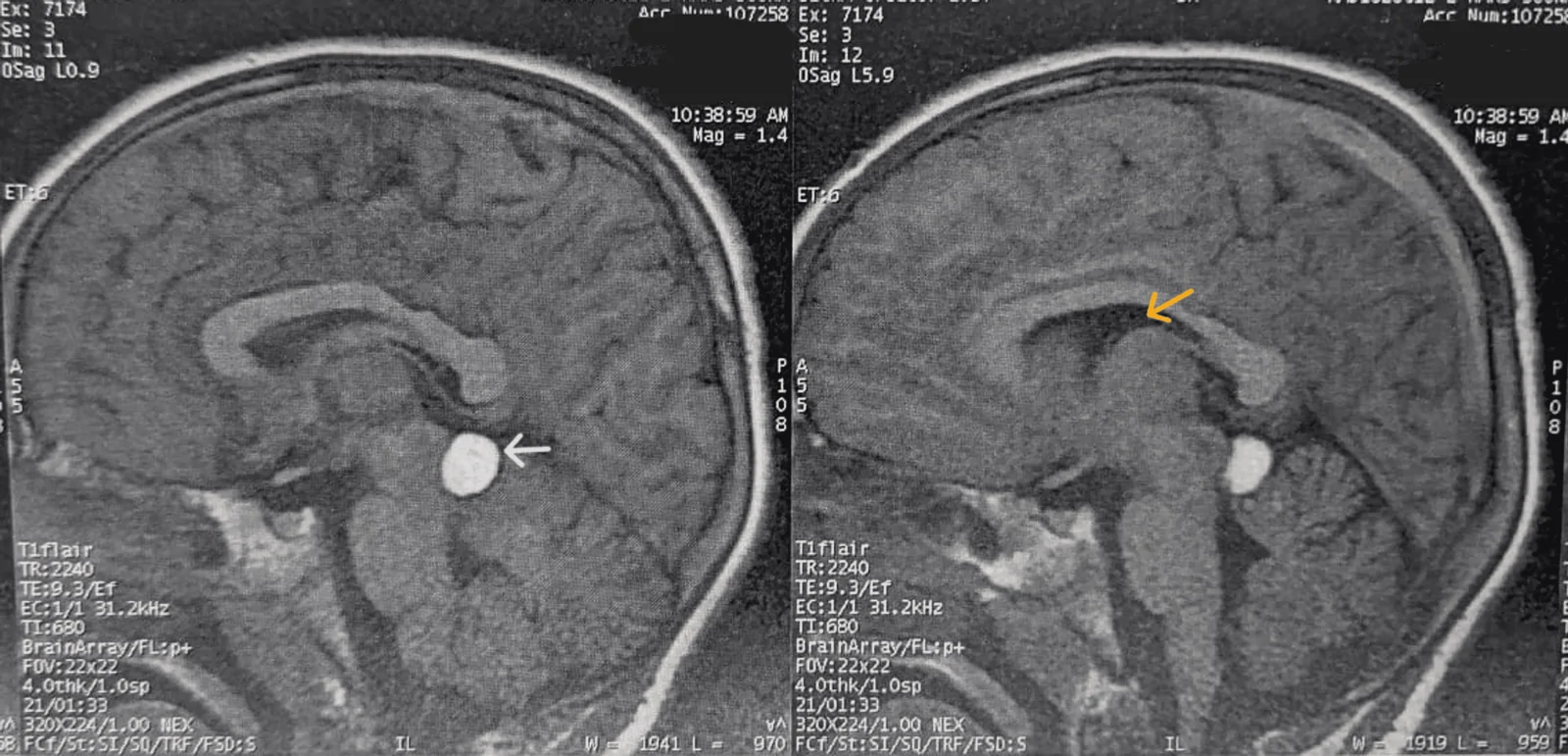
Sagittal fluid-attenuated inversion recovery images magnetic resonance imaging brain image showing lesion in the quadrigeminal cistern (white arrow) without upstream hydrocephalus (yellow arrow)

An 11-year-old child presented following head trauma, with no other associated symptoms. His medical history was unremarkable, and the neurological examination was normal. An emergency cranial CT scan was performed as the initial imaging modality, demonstrating a lesion at the level of the cerebral aqueduct. Further evaluation with brain MRI allowed more detailed characterization of the lesion. It revealed a nonenhancing 1.3-cm right paramedian fat-intensity mass consistent with a lipoma in the quadrigeminal plate cistern, with extension into the aqueduct of Sylvius without causing hydrocephalus. It was hypointense on T1 fat-saturated images (Figure 1) and hyperintense on T2-weighted images (Figure 2), compressing the brainstem anteriorly and the vermis posteriorly (Figure 3). The team chose a conservative management approach. 

## Discussion

Intracranial lipomas are rare developmental abnormalities that originate from defective differentiation of the primitive meningeal tissue during embryogenesis rather than from true neoplastic proliferation. These lesions represent a very small proportion of intracranial masses and are usually identified incidentally during neuroimaging performed for unrelated reasons. Their congenital origin explains their frequent association with anomalies involving adjacent cerebral structures and vascular anatomy [1,4].

Among the different anatomical locations, the pericallosal region remains the most commonly affected site, whereas lesions arising within the quadrigeminal cistern are considerably less frequent. Extension toward or into the aqueduct of Sylvius is exceptionally uncommon and has only rarely been documented in the literature. The present observation therefore illustrates an unusual topographical presentation of an intracranial lipoma in a pediatric patient [4,5].

Most quadrigeminal cistern lipomas are diagnosed in children or young adults and generally follow a benign clinical course. In many situations, the lesion is discovered incidentally after imaging performed for headaches, trauma, or nonspecific neurological complaints. Our patient reflects this common scenario, as the lesion was identified following minor cranial trauma in the absence of neurological deficits or signs of intracranial hypertension. Similar incidental presentations have been described in previously published case reports [1,6].

Magnetic resonance imaging is considered the reference examination for establishing the diagnosis. The typical radiological appearance includes marked hyperintensity on T1-weighted sequences, variable hyperintensity on T2-weighted images, absence of enhancement after gadolinium injection, and signal suppression on fat-saturated sequences. These imaging findings are generally sufficient to confirm the lesion's lipomatous nature and differentiate it from other lesions of the quadrigeminal region. In our case, MRI demonstrated a small right paramedian lipoma occupying the quadrigeminal cistern with extension into the Sylvian aqueduct, without associated ventricular enlargement. Although a mild mass effect on the brainstem and vermis was noted, no clinical repercussions were observed.

The clinical manifestations of quadrigeminal plate lipomas are highly variable and mainly depend on lesion size, the degree of local compression, and the presence of cerebrospinal fluid obstruction. While most patients remain asymptomatic, some authors have reported symptoms including headaches, epilepsy, diplopia, cranial nerve dysfunction, vertigo, psychiatric manifestations, and hydrocephalus related to aqueductal compression [2,6,7]. Fagundes et al. reported a pediatric case complicated by hydrocephalus and seizures requiring surgical treatment, whereas Sehgal et al. described visual hallucinations associated with a tectal lipoma [2,7]. These observations demonstrate the broad spectrum of possible presentations despite the benign histological nature of the lesion.

Therapeutic management remains predominantly conservative. Surgical excision is generally avoided because these lesions are frequently adherent to critical neurovascular structures, particularly within the deep midline cisterns. Attempting complete resection may therefore expose the patient to significant neurological morbidity, including vascular injury or brainstem damage. Consequently, surgery is usually limited to selected symptomatic patients presenting with progressive hydrocephalus, refractory epilepsy, or severe neurological deterioration [1,2,4].

In the present case, the absence of hydrocephalus, neurological symptoms, or radiological progression justified a nonoperative approach with regular clinical and radiological monitoring. This strategy is supported by most published studies, which emphasize the favorable long-term evolution of asymptomatic intracranial lipomas and the limited benefit of aggressive surgical intervention.

## Conclusions

Quadrigeminal cistern lipoma with extension into the aqueduct of Sylvius is an exceptionally rare congenital intracranial malformation that is usually discovered incidentally and remains asymptomatic. MRI plays a crucial role in establishing the diagnosis and evaluating potential complications, particularly local mass effect and obstructive hydrocephalus.

Given its benign nature, slow growth, and close relationship with critical neurovascular structures, conservative management with clinical and radiological follow-up is the preferred approach in asymptomatic patients. The present case supports current evidence favoring observation over surgery, reserving intervention for patients who develop significant neurological symptoms or progressive hydrocephalus.
